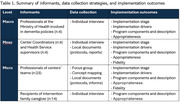# Implementation analysis of community‐based centers for people with dementia and their family caregivers in Chile

**DOI:** 10.1002/alz.093251

**Published:** 2025-01-09

**Authors:** Jean Gajardo, Josefina Aubert, Ximena Moreno, Jose M Aravena, Felipe Carcamo, Maria T Abusleme, Carolina Barrientos, Franco Mascayano

**Affiliations:** ^1^ Universidad San Sebastian, Santiago, Santiago Chile; ^2^ Millennium Institute for Care Research (MICARE), Santiago, Santiago Chile; ^3^ Yale University, New Haven, CT USA; ^4^ US Embassy, Santiago, Santiago Chile; ^5^ New York State Psychiatric Institute, new york, NY USA; ^6^ Columbia University, new york, NY USA

## Abstract

**Background:**

Community‐based centers for people living with dementia (PLWD) have been implemented as part of the National Plan of dementia in Chile, delivering a multicomponent intervention to support PLWD and their families. There is limited knowledge on their implementation, and we had previously informed with respect to the implementation outcomes of the first center, called Kintun. This work offers an update on findings describing implementation outcomes of three more centers, with different implementation times and geographic locations in Chile.

**Method:**

We conducted a realistic evaluation using multiple qualitative data collection strategies and diverse informants to analyze complex interventions considering 4 centers. For every center, key informants were identified at different levels (macro‐level of centralized policy, meso‐level of center coordinators and health service supervisors, and micro‐level of intervention deliverers and family caregivers) who participated in individual interviews or focus groups. Table 1 summarizes informants, data collection strategies, and implementation outcomes. Rapid qualitative analysis was used with predetermined codes describing implementation outcomes according to the Active Implementation Frameworks (Blanchard et al, 2017) and implementation outcomes by Proctor et al (2011).

**Result:**

At a macro‐level of implementation, the centers have not experienced relevant transformations, but the lack of updated centralized technical orientations menace fidelity. At a meso and micro level, the centers have been adapted to improve sustainability and appropriateness, supporting their scalability. Differences in core components characterize a transition from adult day services to a multicomponent intervention, following a progressive interprofessional approach. Examples of implementation drivers were engagement with the local health network and and tailoring the intervention according to geographical and cultural context. Relevant implementation aspects of agreement and disagreement among actors were the appropriateness of the program and the program components, respectively.

**Conclusion:**

Most relevant implementation drivers and adaptations to community centers for PLWD in Chile are identified at micro and meso levels, which stresses the need to define intervention components for standardization and monitoring, providing an implementation frame for new centers. Points of agreement and disagreement among the three levels are key for understanding priorities for improvement